# 1.5 °C pathways for the Global Industry Classification (GICS) sectors chemicals, aluminium, and steel

**DOI:** 10.1007/s42452-022-05004-0

**Published:** 2022-04-01

**Authors:** Sven Teske, Sarah Niklas, Simran Talwar, Alison Atherton

**Affiliations:** Institute for Sustainable Futures, University of Technology Sydney (UTS), 235 Jones Street, Sydney, NSW 2007 Australia

**Keywords:** 1.5 °C pathways, Industry sectors, Decarbonisation for GICS Classified sectors, Chemical industry, Aluminium industry, Steel industry

## Abstract

To achieve the goals of the Paris Climate Agreement, decarbonization targets and benchmarks for specific industry sectors are required. This opens up a whole new research area for energy modelling because although decarbonization pathways have been developed for countries, regions, or communities, few have been developed for industry sectors. In this research, we document the development of energy scenarios for industry sectors classified under the Global Industry Classification Standard (GICS). A bottom-up energy demand analysis based on market projections for the chemical, aluminium, and steel industries forms the basis for scenario development, with the aim of completely decarbonizing the electricity and process heat supplies for these industries by 2050. We document the individual steps in the energy demand analyses based on industry-specific market projections and energy intensities. In the last step, the carbon budget is calculated. The complete decarbonization of the industries analysed seems possible based on the available technology.

**Supplementary Information:**

The online version contains supplementary material available at 10.1007/s42452-022-05004-0.

## Article highlights

- 1.5 °C Pathways for specific industries classified with Global Industry Classification System (GICS)
- Net-Zero decarbonisation pathways for the chemical industry, aluminium industry, steel industry, forestry, and processing harvested products
- Role of database for industry specific energy intensities and energy demand and supply statistic

**Supplementary Information:**

The online version contains supplementary material available at 10.1007/s42452-022-05004-0.

## Introduction

Achieving the goals of the Paris Climate Agreement will require the total decarbonization of the global energy system by 2050, with an emissions peak between 2020 and 2025 [1], and a drastic reduction in non-energy-related greenhouse gases (GHGs), including land-use-related emissions [2]. The global finance industry is taking increasing climate responsibility and are reviewing their investment portfolios in terms of embedded carbon and the extent to which their assets are consistent with the Paris Climate Agreement, which has been ratified by 197 countries [3].

The United Nations Climate Change *Race to Zero Campaign* aims to mobilize cities, regions, businesses, financial institutions, and higher education institutions to establish ‘net-zero’ decarbonization targets outside national governments [4]. To develop, implement, and monitor those net-zero targets, tailor-made energy scenarios for the various actors are required. The finance industry, for example, needs energy and emissions pathways for specific industries, such as the steel or aluminium industry. The International Energy Agency published ‘Net-Zero by 2050—A Roadmap for the Global Energy Sector’ [5] to support net-zero target setting. However, more-detailed energy scenarios, with higher resolution and broken down by industry sub-sectors, are required for institutional investors. The UN-convened Net-Zero Asset Owner Alliance is an international group of institutional investors committed to transitioning the members’ investments to net-zero emissions by 2050 [6]. The Institute for Sustainable Futures (ISF) at the University of Technology (UTS; Sydney, Australia) was invited to develop science-based energy and GHG emissions strategies for specific industries.

The project included various industry sectors. However, in this article, we focus on three major industry sectors—the aluminium, steel, and chemical industries—that produce key primary products to supply almost all other industries. Those three industries are not only relevant to the global economy, with a 19% share of all global industry services, but account for significant shares of the global energy demand. In this research, we developed sectorial 1.5 °C pathways to decarbonize the energy supply for energy-intensive industries. The underlying energy scenario for this research is an updated global 1.5 °C pathway that was first published in 2019 [7].

Compared with other research, the novelty and contributions of this study are as follows:

- It introduces a novel methodology and framework in which detailed industry-sector energy scenarios, based on the GICS classification system, are developed as part of an interconnected energy system model, rather than as separate isolated scenarios. The interconnection of all the industry-specific scenarios allows the development of energy and emissions pathways that reflect sector coupling and possible synergistic effects.
- It provides technical and economic details of the energy scenarios for three energy-intensive industries (chemicals, steel, and cement). The economic prediction pathways—in terms of GDP development and product volume—were developed in dialogue with the respective industries. The industry-specific energy intensities for power and heat demand are further broken down into four temperature levels. The paper provides important input data for future energy scenarios.
- It defines an industry-specific carbon budget for the main industry, in terms of both the annual energy-related CO_2_ emissions and the total cumulative CO_2_ budget, to achieve an increase in the global temperature of + 1.5 °C with 67% likelihood, as defined by the Intergovernmental Panel on Climate Change [1].

The paper is organized as follows. Section 2 presents the Global Industry Classification Standard (GICS) and the role it plays in achieving net-zero targets. Section 3 provides an overview of the industries analysed (chemical, steel, and cement) in terms of the structure of the industry; the main production and/or manufacturing processes; the assumed GPD development based on the reference year 2019, with projection for 2025, 2030, 2035, 2040, and 2050; and the current and projected energy intensities. Section 4 presents the calculated results for all three industries of their final energy demands and energy supplies, further broken down into electricity and process heat generation, and the resulting energy-related CO_2_ emissions, until 2050. Section 5 discusses the conclusions drawn and the further research required.

## Role of the Global Industry Classification Standard (GICS) in achieving net-zero targets

Investment decisions, such as the decarbonization target of the Net-Zero Asset Owner Alliance, are highly complex processes. In November 2020, the European Central Bank published a *Guide on climate-related and environmental risks*, which maps out a detailed process for undertaking “climate stress tests” for investment portfolios. To achieve the Paris Climate Agreement goals in the global finance industry, decarbonization targets and benchmarks for industry sectors are required. This opens up a whole new research area for energy modelling because although decarbonization pathways have been developed for countries, regions, or communities, few have been developed for industry sectors. The OneEarth Climate Model (OECM) is an integrated assessment model for climate and energy pathways that focuses on 1.5 °C scenarios [7], and has been further improved to meet this need. To develop energy scenarios for industry sectors classified under the Global Industry Classification Standard (GICS), the technological resolution of the OECM required significant improvement. Furthermore, all demand and supply calculations had to be broken down into industry sectors before the individual pathways could be developed.

The Global Industry Classification Standard (GICS) was developed by the American investment research firm Morgan Stanley Capital International (MSCI) and Standard & Poor’s (S&P), a finance data and credit ratings company, in 1999. According to MSCI, the GICS was designed to define specific industry classifications for reporting, comparison, and investment transaction processes [8]. The GICS has four classification levels and includes 11 sectors, 24 industry groups, 69 industries, and 158 sub-industries. The 11 GICS sectors are: Energy, Materials, Industrials, Consumer Discretionary, Consumer Staples, Health Care, Financials, Information Technology, Communication Services, Utilities, Real Estate.

Table 1 provides an overview of the Materials sector (1510) and all sub-sectors. In this analysis, we focused on the sub-sectors chemical (1510 10), aluminium (1510 4010), and steel (1510 4050).

**Table 1:** Global Industry Classification Standard (GICS)—materials sector

1510 Materials	
*1510 10*	*Chemicals*
	*15101010 commodity chemicals*
	*15101020 diversified chemicals*
	*15101030 fertilizers & agricultural chemicals*
	*15101040 industrial gases*
	*15101050 specialty chemicals*
1510 20	Construction materials
	1510 2010 construction materials
1510 30	Containers & packaging
	1510 3010 metal & glass containers
	1510 3020 paper packaging
1510 40	Metals & mining
	*15104010 aluminium*
	1510 4020 diversified metals & mining
	1510 4025 copper
	1510 4030 gold
	1510 4040 precious metals & minerals
	1510 4045 silver
	*15104050 steel*
1510 50	Paper & forest products
	1510 5010 forest products
	1510 5020 paper products

The individual industries are briefly described below. The focus here is exclusively on current and future market developments and the energy-related aspects of the production of these products. The development of the market for aluminium and steel was based on the material throughput in tons per year. This was not possible for the chemical industry due to the diversity of products and therefore the market development was calculated on the basis of GDP.

## Aluminium, steel, and chemical industries and their direct shares of the global gross economic product

The global gross domestic product (GDP) in 2019 was US$87.8 trillion, 3% of which came from agriculture, 26% from industry, 15% from manufacturing, and the remaining 65% from services [9]. The aluminium and steel industries each had a 1% direct share of the global industry GDP value and the chemical industry’s share was 17%, although the indirect effects of those industries are significantly higher. The materials produced by these three industries are essential for the manufacturing and service industry, which generate 80% of the global GDP. In the next section, the status quo of the aluminium, steel, and chemical industries is briefly described.

The OECM is an integrated energy assessment model that covers the entire global energy system, broken down into various sub-sectors. In this paper, we focus on the aluminium industry, steel industry, and chemical industry. However, the projections for the economic development for these three industries are developed in the context of the overall global socio-economic development—namely, the development of the population and the projected GDP until 2050. Furthermore, the industry-specific projections (such as the development of production volumes in million tonnes of steel and aluminium) are based on the results of workshops with industry, academia, and the NZAOA Scientific Advisory Board (UNEPFI 2021) organized by the Net-Zero Asset Owner Alliance (NZAOA) between May 2020 and November 2021.

### Global chemical industry (1510 10) overview

The chemical industry is an important intermediate industry, which is engaged in the conversion of raw materials, such as fossil fuels, minerals, metals, and water, into a variety of chemical products used in industrial sectors, including pharmaceuticals, fertilizers, pesticides, plastics, dyes, paints, and consumer products. Close overlaps exist between the chemical and plastic industries, with many chemical producers also involved in plastics manufacture. Revenue from the global chemical industry increased by 48% to US$3.9 trillion between 2005 and 2019 [10, 11]. With 26.4% Pharmaceuticals have the largest share in the segment-wise breakdown of global chemical shipment in 2019, followed by bulk petrochemicals and intermediates (16.4%), specialties (16%, plastics resins (12.2%), agricultural chemicals (8.6%), consumer products (8.3%), inorganic chemicals (7.1%) manufactured fibre (4%) and synthetic rubbers (1%). Together, the world’s 100 leading chemical companies generated US$1.05 trillion in revenue in 2019 [12]. Although the ownership of chemical industry production has changed significantly over the past two decades, the shares of feedstocks for chemical production—gas liquids, naphtha, oil, natural gas, and coal—remained stable between 2000 and 2020 (UNEP, 2019).

Basic organic and inorganic chemicals account for the highest shares of production and consumption (by volume) in the global chemical industry [13].

- **Basic chemicals**, also known as ‘commodity chemicals’, consist of both organic and inorganic chemicals that are used as feedstock materials for a variety of downstream chemicals. Some of the most frequently used basic chemicals are methanol, olefins (such as ethylene and propylene), and aromatics (such as xylenes, benzene, and toluene). Basic chemical production processes are well established, with high capital and energy demands. Among these basic chemicals, petrochemicals and their derivatives, such as organic intermediates, plastic resins, and synthetic fibres, are strongly traded commodities, with ethylene, propylene, and methanol production capacities accounting for a vast share of petrochemical production globally.
- **Inorganic chemicals** include acids and bases, salts, industrial gases, and elements such as halogens. Inorganic chemicals are used as intermediate inputs in the manufacture of many specialty chemicals, such as solvents, coatings, surfactants, electronic chemicals, and agricultural chemicals. Nitrogen compounds account for the largest share of inorganic chemical production globally. With the rise in glass and paper production, the demands for soda ash and caustic soda are increasing rapidly, coupled to the high demand for inorganic chemicals in the food and cosmetics industries.

#### Major chemical industry companies and countries

BASF (headquarters [HQ] in Germany), Dow (HQ USA), and Sinopec (HQ China) were some of the world’s largest chemical-producing companies (based on sales) in 2018. Each of these three leaders exceeded US$65,000 million in chemical sales. Eighteen countries were represented in the list of the top 50 chemical companies in 2019, and more than 50% of them were headquartered in the USA (10), Japan (8), and Germany (5) [14]. German companies BASF, Bayer, and Linde are the foremost international producers. BASF, for example, owns global operations in the chemical industry and is active across the entire value chain, spanning the manufacture of chemicals, plastics, performance products, functional and agricultural solutions, oil, and natural gas. Bayer is a well-known pharmaceutical and chemical manufacturer, and the Linde group owns large industrial gas and engineering facilities, which produce various gas products, such as atmospheric oxygen, nitrogen, and argon.

#### Chemical manufacturing and energy intensity

The chemical industry uses raw materials from natural gas, ethane, oil-refining by-products (such as propylene), and salt to manufacture bulk chemicals, such as sulfuric acid, ammonia, chlorine, industrial gases, and basic polymers, including polyethylene and polypropylene. The manufacturing activity within the chemical industry can be divided into two main categories: basic chemicals and chemical products.

Basic chemicals are those chemicals that feed into the manufacture of other complex chemicals. Petroleum and coal products can be considered basic chemicals because they are used in the manufacture of a variety of polymers, fibres, and other chemicals. The manufacturing processes for basic chemicals, including inorganic chemicals, organic chemicals (such as ethylene and propylene), and agricultural chemicals, are considered energy-intensive industries and require large production facilities.

The second category involves the manufacture of ammonia, polyethylene, and other chemical products. Ammonia production is an energy-intensive process and is considered to be an important contributor to the chemical industry’s energy and emissions footprints. Ammonium nitrate is used as an agricultural fertilizer and as a blasting explosive in the mining industry. Polyethylene, a by-product of the petrochemical industry, is produced from ethane feedstock, and has a variety of uses in the plastics industry. All other chemical products, such as pharmaceuticals, cleaning products and detergents, cosmetics, paints, pesticides and herbicides, fertilizers, and plastic and rubber products, mainly require non-energy-intensive manufacturing processes. For example, the manufacture of pharmaceuticals, paints, and coatings is classified as non-energy-intensive, owing to the differences in manufacturing processes and technologies within the industry [15]. Production facilities range from small to large enterprises, with energy supplied by either gas or electricity.

#### Chemical industry: sub-sectors chosen for the OECM analysis

To prepare the decarbonization pathways, we have broken down the chemical industry into the following sub-sectors. These sub-sector classifications are based on the main application industries for chemical feedstocks and follow the categorization based on the American Chemistry Council [12].

1. Pharmaceutical industry
2. Agricultural chemicals
3. Inorganic chemicals and consumer products
4. Manufactured fibres & synthetic rubber
5. Bulk petrochemicals & intermediates, plastic resins

The most important raw materials and chemical products of those five chemical industry subgroups are described below. The division into these sub-groups was based on the available economic data required for the market projection. A market development assessment on the basis of the material flow would be more precise, but was outside of the research scope due to the variety of products produced by the chemical industry. The analysis focused on the development of the chemical industry's energy requirements.

##### Sub-sector 1: Pharmaceuticals

*Products and materials* There are two key stages in pharmaceutical production: (i) the manufacture of the active pharmaceutical ingredients (APIs); and (ii) the production of formulations. An API is the part of the drug that generates its effect. The production of APIs is usually chemically intensive, involving reactors specific for the manufacture of drug substances. Formulation production is a physical process, in which substances known as ‘excipients’ are combined with APIs to create consumable products (tablets, liquids, capsules, creams, ointments, and injectables).

*Production and processes* The world’s largest pharmaceutical companies are headquartered in the USA and Europe, although production activities are centred in Asia. Some of the biggest pharmaceutical companies are Pfizer (USA), Roche, Novartis (Switzerland), Merck (USA), and GlaxoSmithKline (UK). Until the mid-1990s, the USA, Europe, and Japan supplied 90% of the world’s demand for APIs. However, China’s low-cost manufacturing sector and weak environmental regulations have meant that a significant portion of API production has now shifted, with almost 40% of all APIs currently supplied by China. Together, China and India supply almost 75% of the API demand of pharmaceutical manufacturers in the USA. China’s dominance in API production is balanced by India’s leadership in global formulation production and its biotechnology sector. India is also the third largest producer of pharmaceuticals, by volume, supplying most of Africa’s demand. India hosts the highest number of United States Food and Drug Administration (US FDA)-sanctioned production facilities outside the USA, and supplies 40% of the US generic drug market. Despite India’s vast pharmaceutical manufacturing industry, the country still imports 70% of its API demand from China.

*Uses and applications* Pharmaceutical products primarily service the health-care sector, with prescription and over-the-counter drugs, vaccines, and other pharmaceutical applications for human and veterinary use. The biotechnological production of crop seeds, value-added grains, and enzymes is a rapidly growing segment of the industry.

##### Sub-sector 2: agricultural chemicals

*Products and materials* Agricultural chemicals are a type of specialty chemical, and the term refers to a broad variety of pesticide chemicals, including insecticides, herbicides, fungicides, and nematicides (used to kill round worms). Agrichemicals can also include synthetic fertilizers, hormones, and other chemical growth agents, as well as concentrated varieties of raw animal manure [16]. The main raw materials for nitrogen fertilizers are natural gas, naphtha, fuel oil, and coal, whereas phosphate fertilizers are based on naturally occurring phosphate rocks or synthetic ammonia.

*Production and processes* Some of the large agrichemical chemical producers are Syngenta, Bayer Crop Science, BASF, Dow AgroSciences, Monsanto, and DuPont. The fertilizer industry is structured around a few producers who supply the base chemicals to downstream manufacturers. The production facilities usually specialise in single-nutrient or high-nutrient fertilizer products and are located in close proximity to raw material suppliers (petrochemical producers) or agricultural regions [17].

*Uses and applications* Unsurprisingly, large-scale farming, also referred to as ‘industrialized agriculture’, is one of the primary users of agrichemicals. In 2010–2011, the global demand for primary plant nutrients was 178 Megatons (Mt). China (57 Mt), the USA (20 Mt), and India (28 Mt) were the highest consumers.

##### Sub-sector 3: Inorganic chemicals and consumer products

*Products and materials* Inorganic chemicals are materials derived from metallic and non-metallic minerals, such as ores or elements extracted from the earth (e.g., phosphate, sulfur, potash), air (e.g., nitrogen, oxygen), and water (e.g., chlorine). Other examples include aluminium sulfate, lime, soda ash (sodium carbonate), and sodium bicarbonate. The outputs of the chemical industry are used in the manufacture of consumer products, such as soaps, detergents, bleach, toothpaste and other oral hygiene products, and personal care products, such as hair care, skin care, cosmetics, and perfumes.

*Production and processes* Basic chemicals are typically produced in large-scale capital-intensive facilities with high energy demands. Industrial gases, which are also products of the inorganic chemical industry, are heavily used in the production processes associated with steel, other chemicals, electronics, and health care. Many global factors influence the production of industrial gases. These factors include high capital intensity, increased consolidation of operations and geographic concentration, service orientation, and innovations in key technologies, such as membrane separation. The chemical conversion processes for consumer products are basic, and the key raw materials include fats, oils, surfactants, emulsifiers, other additives, and basic chemicals. Consumer products are usually formulated in batch-type operations, which involve equipment for mixing, dispersing, and filling [12].

*Uses and applications* The applications for inorganic chemicals are diverse. For example, chlorine is an important ingredient used to bleach paper pulp and purify drinking water; oil refining and steel industry uses; and caustic soda is used in the production of soaps and detergents. Consumer products are heavily dependent upon vast distribution channels and product segmentation. Therefore, the supply chain and marketing costs are important determinants of product pricing, exacerbated by the need for ongoing product development.

##### Sub-sector 4: manufactured fibres and synthetic rubber

*Products and materials* Manufactured fibres, also referred to as ‘synthetic fibres’, consist of *cellulosic fibres*, such as acetate and rayon, and petrochemical-derived *polymeric fibres*, such as acrylics, nylon, polyesters, and polyolefins. There are several types of synthetic rubber, including butyl rubber, ethylene–propylene–diene monomer terpolymers, neoprene, nitrile rubber, styrene-butadiene rubber, and specialty elastomers [12].

*Production and processes* Synthetic or artificial fibres are derived from polymer industries using processes such as wet spinning (rayon), dry spinning (acetate and triacetate), and melt spinning (nylons and polyesters). Synthetic rubbers have highly flexible material characteristics, and the process of ‘vulcanization’ is used to cross-link elastomer molecules.

*Uses and applications* Plastics, synthetic rubber, and manufactured fibres account for the second highest share (30%) of total energy consumption by the chemical industry in the USA, preceded by petrochemicals and other basic chemicals, which have a 49% share [12]. Synthetic fibres are heavily used in apparel, home furnishings, and the automotive and construction industries. Similarly, synthetic rubber is in high demand in automotive manufacturing, construction, and consumer products. Synthetic fibres are increasingly used in textile manufacture because of their durability and abundance, and their ability to be processed into long fibres or to be batched and cut for processing like natural fibres. Natural fibres, such as wool, silk, and leather, are most frequently used for high-quality and long-lasting garments, whereas synthetic fibres are popular in the manufacture of fast-fashion garments and accessories [18].

##### Sub-sector 5: petrochemicals

*Products and materials* Petrochemicals are chemical products derived from petroleum refining and derived from other fossil fuels, such as natural gas and coal. The two main classes of petrochemicals are olefins and aromatics. Ethylene, propylene, and butadiene are examples of olefins—ethylene and propylene are used in the manufacture of industrial chemicals and plastic products, whereas butadiene is used to manufacture synthetic rubber. Olefins also form the base compounds in the manufacture of the polymers and oligomers used in plastics, resins, fibres, elastomers, lubricants, and gels.

Benzene, toluene, and xylene isomers are examples of aromatic compounds, which are primarily produced from naphtha derived from petroleum refining. Benzene is used as a raw material in the manufacture of dyes and synthetic detergents, whereas xylene is used to manufacture plastic products and synthetic fibres.

Apart from olefins and aromatics, other chemical products of the petrochemical industry include synthetic gases used to make ammonia and methanol (steam-reforming plants), methane, ethane, propane, and butanes (natural-gas-processing plants), methanol, and formaldehyde. Ammonia is also used in the manufacture of the fertilizer urea, whereas methanol is used as a solvent and chemical intermediate.

Globally, 190 million tonnes (Mt) of ethylene and 120 Mt of propylene were produced in 2019, and approximately 70 Mt of aromatics were produced.

*Production and processes* The USA and Western Europe are home to the world’s largest petrochemical producers. Some of the notable petrochemical manufacturing locations are in the industrial cities of Jubail and Yanbu in Saudi Arabia, Texas and Louisiana in the USA, Teesside in the UK, Rotterdam in the Netherlands, and Jamnagar and Dahej in India. The Middle East and Asia are witnessing increasing investment in new production capacities for petrochemical plants, and a vast majority of the global demand is expected to be met from these regions in the coming decade [19]. Some of the fastest-growing petrochemical companies in terms of capacity are PetroChina, Reliance, SABIC, Sinopec, and Wanhua. Both olefins and aromatics can be produced during oil refining by the fluid catalytic cracking of petroleum fractions or with chemical processes. In chemical plants, the process of steam cracking is used to produce olefins from natural gas liquids, such as ethane and propane. For the production of aromatics, a naphtha catalysis process is used.

*Uses and applications* The petrochemical sector supplies materials for the vast majority of chemical industry applications, such as the manufacture of petrochemical derivatives, aromatics from bulk petrochemicals, olefins, and methanol. Seven petrochemicals supply more than 90% of all organic chemicals: benzene, toluene, and xylene (aromatics), ethylene, propylene, and butadiene (olefins), and methanol [12]. Bulk petrochemicals are also transformed into intermediate products and downstream derivatives, such as plastic resins, synthetic rubbers, manufactured fibres, surfactants, dyes, pigments, and inks. The end-user industries for petrochemical products are the chemical industry, automotive industry, building and construction, consumer products, electronics, furniture, and packaging.

#### GDP projections for the global chemical industry

The economic development of the global chemical industry is significantly more complex than that of the aluminium and steel industries. The product range of the chemical industry is diverse, and the material flow approach used for aluminium and steel is very data intensive, so they are beyond the scope of this research. The chemical industry produces materials for almost all parts of the economy—from mining to services—and it is therefore intrinsically connected to overall economic development. Consequently, a GDP-based approach has been used to develop the energy demand projections for the chemical industry over the next three decades.

The United Nation’s Global Chemical Outlook estimates that the feedstock shares for chemical production will remain stable until 2040 as ‘several of the world’s largest chemical producers are owned by fossil fuel producers’ (UNEP, 2019). Based on this, the OECM 1.5 °C pathway for the chemical industry assumes that the five sub-sectors of the chemical industry (described in 3.1.3) will remain stable. A more in-depth analysis of the chemical industry by sub-sector and possible economic development trajectories was beyond the scope of this research.

Table 2 provides an overview of the projected economic development of the chemical industry and its five sub-sectors. It is assumed that the chemical industry will follow the trajectory of global GDP growth, and that the chemical industry’s share of global GDP will remain constant until 2050. The sub-sectors are assumed to grow at the same rate as the overall chemical industry and the market value share of each sub-sector will also remain stable. For example, the pharmaceutical industry had a 26% share of the global chemical industry GDP, just over US$1 billion, in 2019. With this approach, we assume that the share of 26% will remain constant until 2050 and that the growth rate of each sub-sector will develop in line with the global GDP projection. This is a simplification, and the actual development trajectories may vary across all sectors. However, a more nuanced projection of the development of the chemical industry is beyond the scope of this research.

**Table 2:** Projected economic development of the chemical industry [9, 12]

Parameter	[Unit]	2019	2025	2030	2035	2040	2050
Global GDP	[bn $ GDP]	129,555	142,592	196,715	231,758	266,801	346,236
Total chemical industry	[bn $ GDP]	3900	4966	5862	6906	7950	10,317
Global GDP share	[%]	3%	3%	3%	3%	3%	3%
pharmaceutical industry	[bn $ GDP]	1029	1314	1551	1828	2104	2730
GDP—share of the chemical industry market value	[%]	26%	26%	26%	26%	26%	26%
Agricultural chemicals	[bn $ GDP]	333.8	424.5	501.1	590.4	679.6	882.0
GDP—share of the chemical industry market value	[%]	9%	9%	9%	9%	9%	9%
Specialties, inorganic chemicals, consumer products	[bn $ GDP]	1225	1558	1839	2167	2495	3237
GDP—share of the chemical industry market value	[%]	31%	31%	31%	31%	31%	31%
Manufactured fibres & synthetic rubber	[bn $ GDP]	196.6	249.9	295.0	347.6	400.2	519.3
GDP—share of the chemical industry market value	[%]	5%	5%	5%	5%	5%	5%
Bulk petrochemicals & intermediates, plastic resins	[bn $ GDP]	1115.8	1418.9	1674.9	1973.2	2271.6	2947.9
GDP—share of the chemical industry market value	[%]	29%	29%	29%	29%	29%	29%

#### Energy flows for the chemical industry

Natural gas and petroleum products are important energy sources for the chemical industry. Globally, the chemical industry is responsible for 11% of the primary demand for oil and 8% of the primary demand for natural gas [20]. The chemical industry in the USA consumes almost 9% of all petroleum products as feedstock for fuel and power use, natural gas liquids (or liquefied petroleum gases), and heavy liquids (naphtha and gas oil) [12].

*Petrochemical feedstocks*, such as olefins and aromatics, are extracted from hydrocarbons produced with cracking processes. These feedstocks are used in the plastics, pharmaceuticals, electronics, and fertilizer industries. *Methanol* is directly converted from the methane in natural gas and does not undergo the cracking process. In the USA, natural gas liquids are used in the production of 90% of olefins, whereas naphtha is the main source (70%) of petrochemical production in Europe and Asia.

IEA [21] mapped the flows of fuel feedstocks in the chemical and petrochemical industries in 2015. Most of the oil feedstock was converted to high-value chemicals, and a large proportion of raw materials for the chemical industry were directly supplied from oil refineries. Ammonia and methanol, both chemicals in high demand, require natural gas as the raw material. China also uses coal in the production of ammonia and methanol. Petrochemical production occurs in very large-scale facilities, and a number of related products can be produced at a single petrochemical facility. This differs to the set-ups for commodity chemicals, where specialty chemicals and fine chemicals are manufactured in discrete batch processes. Historically, the accelerating demand for chemical products in these end-use industries has had an inevitable impact on the energy demand and resultant CO_2_ emissions of the upstream and overall chemical industry. Together, base chemicals supply the intermediate raw materials for the majority of the aforementioned demand industries [20, 21].

The energy demand in the pharmaceutical industry is largely driven by the critical environmental requirements for temperature, humidity, room pressurization, cleanliness, and containment. The manufacturing and R&D phases consume a high proportion of the energy demand (> 65%), followed by the formulation, packaging, and filling phases (15%). Overall, heating, ventilation, and air conditioning are the highest energy end uses in the industry (> 65%), because of the nature of the products manufactured [22]. Another energy-consuming system is the production of compressed air, which has multiple applications and is one of the least energy-efficient functions in a pharmaceutical production facility. There are opportunities for energy and cost savings in this area [22]. In the production of agrochemicals, the energy demand is spread across manufacturing, packaging, and transportation, and the majority of raw materials are derived from the petrochemical industry. The production of nitrogen fertilizers is energy intensive due to the energy-intensive conversion process from the fossil-fuel raw materials used to manufacture the usable fertilizers. In terms of material throughput, one tonne of nitrogen fertilizer output consumes 1.5 tonnes of equivalent petrol [23].

#### Projection of the chemical industry energy demand

The brief overview of the energy usage for the sub-sectors analysed has shown that the chemical industry consists of a highly energy-intensive part that produces the primary feedstocks (basic chemicals) and a secondary product manufacturing part, with a relatively low energy intensity, comparable to those of other manufacturing industries with energy intensities of < 10 MJ per $GDP.

The energy demands for the five sub-sectors—pharmaceuticals, agricultural chemicals, inorganic chemicals and consumer products, manufactured fibres and synthetic rubber, and the petrochemical industry—were calculated with the energy intensities provided in Table 3. The energy intensities are based on the IEA Energy Efficiency extended database [24] and our own research. The energy intensities for primary feedstocks were also considered in estimating the efficiency trajectories of the different sectors. An increase in the efficiency of the primary feedstock production of 1% per year over the entire modelling period is required to achieve the assumed efficiency gains for all sub-sectors. However, few data are available for the specific energy intensities of the chemical industry and no detailed breakdown into the electricity and process heat temperature levels is available in the databases in the public domain. Therefore, our estimates should be seen as approximate values and more research, in co-operation with the chemical industry, is required. However, the energy requirements of the entire chemical industry are precisely known and were taken from the IEA statistic *Advanced Energy Balances* [25]. The energy requirements of the sub-sectors were determined on the basis of market shares and GDP and in discussions with representatives of the chemical industry—specifically members of the Net-Zero Asset Owner Alliance and the Strategic Approach to International Chemicals Management of the United Nations Environmental Program (SAICM UNEP).

**Table 3:** Assumed energy intensities for sub-sectors of the chemical industry

Chemical industries—energy intensities		2019	2025	2030	2035	2040	2050
Pharmaceutical industry	[MJ/$GDP]	5.02	4.54	4.36	4.18	4.02	3.70
Assumed annual increase in efficiency	[%/yr]		0.80%	0.80%	0.80%	0.80%	0.80%
Agricultural chemicals	[MJ/$GDP]	8.37	7.56	7.26	6.97	6.69	6.17
Assumed annual increase in efficiency	[%/yr]		0.80%	0.80%	0.80%	0.80%	0.80%
Inorganic chemicals and consumer products	[MJ/$GDP]	4.22	3.81	3.66	3.51	3.37	3.11
Assumed annual increase in efficiency	[%/yr]		0.80%	0.80%	0.80%	0.80%	0.80%
Manufactured fibres and synthetic rubber	[MJ/$GDP]	4.97	4.49	4.31	4.14	3.97	3.66
Assumed annual increase in efficiency	[%/yr]		0.80%	0.80%	0.80%	0.80%	0.80%
Bulk petrochemicals and intermediates, plastic resins	[MJ/$GDP]	4.64	4.15	3.94	3.75	3.56	3.21
Assumed annual increase in efficiency	[%/yr]		1.00%	1.00%	1.00%	1.00%	1.00%
**Energy intensities**—primary feedstocks							
Petroleum Refining	[MJ/$GDP]	54.16	51.45	48.88	46.44	44.11	39.81
Assumed annual increase in efficiency	[%/yr]		1.00%	1.00%	1.00%	1.00%	1.00%
Alkali and chlorine manufacturing	[MJ/$GDP]	63.85	60.66	57.62	54.74	52.01	46.94
Assumed annual increase in efficiency	[%/yr]		1.00%	1.00%	1.00%	1.00%	1.00%
All other basic inorganic chemical manufacturing	[MJ/$GDP]	54.34	51.62	49.04	46.59	44.26	39.94
Assumed annual increase in efficiency	[%/yr]		1.00%	1.00%	1.00%	1.00%	1.00%
Chemical fertilizer (except potash) manufacturing	[MJ/$GDP]	110.41	104.89	99.65	94.66	89.93	81.16
Assumed annual increase in efficiency	[%/yr]		1.00%	1.00%	1.00%	1.00%	1.00%
Chemical industries—average energy intensity	[MJ/$GDP]	4.36	3.64	3.56	3.49	3.42	3.29
Assumed annual increase in efficiency	[%/yr]		1.00%	1.00%	1.00%	1.00%	1.00%

The global production and energy intensity projections were used to project the energy demand and emissions pathways for all sectors, which are presented in the following sections.

### Aluminium industry (1510 4010)

This section provides an overview of the aluminium industry. Aluminium is among the most important building and construction materials in the world. To understand the opportunities and challenges facing the industry, the global flow of aluminium metal must be considered. Since 1880, an estimated 1.5 billion tonnes of aluminium has been produced worldwide [26], and about 75% of the aluminium produced is in productive use [27]. In 2019, 36% of aluminium was located in buildings, 25% in electrical cables and machinery, and 30% in transport applications. Aluminium can be recycled, but the availability of scrap is limited by the high proportion of aluminium in use [26].

#### Bauxite production

Primary aluminium production requires bauxite. Bauxite ore occurs in the top soils of tropical and subtropical regions, such as Africa, the Caribbean, South America, and Australia. The largest producers/miners of bauxite include Australia, China, and Guinea. Australia supplies 30% of global bauxite production [28]. Table 4 shows the global distribution of bauxite mine production and aluminium refineries and production.

**Table 4:** Aluminium resources, including bauxite mines, alumina refineries, and aluminium production in thousand tonnes (10^3^ t), by country

	Bauxite mine production		Alumina refineries/production	Bauxite reserves	Aluminium production	Aluminium production in %
	2018	2019^a^ (estimated)	2018	No year	2019	2019
Australia	86,400	100,000	20,400	6,000,000	20,000	15.1%
China	79,000	75,000	72,500	1,000,000	73,000	54.9%
Guinea	57,000	82,000	180^c^	7,400,000	No data	No data
Brazil	29,000	29,000	8100	2,000,000	8900	6.7%
India	23,000	26,000	6430	660,000	6700	5.0%
Indonesia	11,000	16,000	1000	1,200,000	No data	No data
Jamaica	10,100	8900	2480	2,000,000	1800	1.4%
Russia	5650	5400	2760	500,000	2700	2.0%
Vietnam	4100	4500	1310	3,700,000	No data	No data
Saudi Arabia	3890	4100	1770	200,000	1800	1.4%
United States	W^a^	W	1570	20,000	1600	1.2%
Canada	No data	No data	No data	No data	1500	1.1%
Other countries	17,000^d^	15,000	11,400^d^	5,000,000^d^	14,600	11.0%
World total	327,000^b^	370,000^b^	131,000	30,000,000	132,900	100%

*Source* (U.S. Geological Survey, 2020)

^a^Estimated. E, net exporter; W, withheld to avoid disclosing company proprietary data

^b^Excludes US production

^c^Only one of the bauxite producers in Guinea refines the raw material in the country; other aluminium refineries are owned by Russian exporters and Chinese operators

^d^Includes Canada

#### Aluminium production

Globally, 63.7 million tonnes of primary aluminium were produced in 2019 (IAI, 2021c). About 32 million tonnes of aluminium is recycled every year [29]. Global primary aluminium production accounts for two thirds of total production. However, not all bauxite-rich countries are among the main aluminium-producing nations. China dominates global aluminium production. Overall, nine conglomerates are responsible for global aluminium production (31.5 million tonnes/year), and of those, four have their headquarters in China ((Statista, 2021): Chalco, Hongqiao Group, Xinfa, and SPIC Aluminum & Power Investment Co. Ltd ((Statista, 2021). As a result, Chinese aluminium companies produce 17.8 million tonnes per year, or 57% of the volume produced by the nine major companies ((Statista, 2021). Russian aluminium manufacturer Rusal produces 3.8 million tonnes annually, which is 12% of the amount produced by the nine largest companies. Like China, Russia also owns an aluminium refinery in Guinea [30]. The Australian/UK mining giant Rio Tinto produces 3.2 million tonnes/year, equivalent to 10.2% of the aluminium produced by the main producers; the UAE aluminium producer EGA produces 2.6 million tonnes/year (8%), the US-owned company Alcoa produces 2.5 million tonnes/year (6.9%); and Norwegian Norsk Hydro produces 2 million tonnes/year, which is equivalent to 6% of aluminium produced by the nine top companies [30]. Another 1.9 million tonnes/year is produced by other companies.

The proportion of recycled or ‘secondary’ aluminium production is a key consideration for decarbonization pathways because secondary aluminium production is up to 95% less energy intensive than its primary production from bauxite [31]. The aluminium sector distinguishes between new aluminium scrap (off-cuts generated during the manufacture of aluminium) and old scrap (used, discarded, and collected aluminium products). The proportion of aluminium that is recycled can be measured by quantifying the input rate and the efficiency rate:

- The recycling input rate describes the proportion of new and old scrap fed into aluminium production.
- The recycling efficiency rate is the proportion of aluminium available that is recovered from a region.

Once collected, the metal losses from recycling processes are usually < 2%, so the net metal yield is > 98% [32]; based on a 2005 study). The global recycling input rate has remained constant, at around 32%, since 2000 [31]. The most recent data show a global recycling input rate of 32% in 2020, whereas in 2018, the global recycling input rate was 33%, and old scrap accounted for 60% of this.

Globally, up to 30 Mt of primary aluminium was recycled in 2020, equivalent to a recycling rate of 76% [31].

- **Europe** has the highest aluminium recycling efficiency rate worldwide, and 81% of scrap available in the region is recovered [31].
- The **USA** has the highest recycling input rate, at 57%.
- **China** is the largest producer and consumer of recycled aluminium; it produces 10 million tonnes of secondary aluminium from scrap annually, or 33% of the global volume [31].

#### Aluminium production processes

An analysis of current and future aluminium production processes is required to understand the decarbonization opportunities within each process.

*Primary aluminium production* involves the following processes (excluding mining):

1. *Refining bauxite to produce alumina (Bayer chemical process)* Bauxite contains ores other than aluminium, including silica, various iron oxides, and titanium dioxide [33]. Alumina, an aluminium oxide compound, is chemically extracted with the Bayer process [34], in which bauxite ore is ground and then digested in highly caustic solutions at elevated temperatures. Approximately 70% of global bauxite production is refined to alumina with the Bayer process [33].
2. *Smelting* is the process of refining alumina to pure aluminium metal (Hall–Héroult electrolytic process). Alumina is dissolved at 950 °C (1750 °F) in a molten electrolyte composed of aluminium, sodium, and fluorine, to lower its melting point, allowing easier electrolysis. An electrical reduction line is formed by connecting several electrolysis cells in series [35]. Electrolysis separates alumina into aluminium metal at the cathode and oxygen gas at the anode [28].

In the *secondary production of aluminium (aluminium recycling process)*, the process of refining the raw material (bauxite) to alumina is not required. Instead, scrap aluminium is re-melted and refined. Therefore, the energy consumption for this process is much lower than for primary production [31, 35].

#### Aluminium industry: Energy demand and energy intensities

The amount of energy used to generate a unit of GDP is referred to as the ‘energy intensity of the economy’ [36]. The IEA analyses the energy intensity for different sectors in the economy per GDP based on the US currency. The energy intensities of primary and secondary aluminium production are reported under the sub-sector ‘basic metals’. In 2018, the production of basic metals was responsible for 27% of the energy consumption in the Manufacturing sector. The sub-sector basic metals, includes ferrous metals (22% of energy consumption) and non-ferrous metals, such as aluminium, nickel, lead, tin, brass, silver, and zinc, and accounts for 5% of the manufacturing sector’s energy consumption [36]. Table 5 below shows the energy intensities of the total basic metal and non-ferrous metal sub-sectors by region.

**Table 5:** Energy intensities (energy consumption per value added) in the manufacturing industry sub-sectors basic metals and non-ferrous metals, by region (2018 data; MJ per GDP in USD 2015), by region

	Energy intensity, 2018 data [MJ/GDP in USD, 2015]		
	Basic metals (ferrous and non-ferrous)	Ferrous metals^a^	Non-ferrous metals^a^
Percentage share of global energy intensity [%]	27	22	5
Global	2724.00	2219.56	504.44
North America [MJ/GDP in USD]	290.00	236.30	53.70
Europe [MJ/GDP in USD]	1568.00	1277.63	290.37

*Source* [36]

^a^Derived calculation from the total energy consumption of the basic metals sector

Compared with aluminium production processes, the energy demand for bauxite mining is relatively small. Bauxite mining requires < 1.5 kg of fuel oil (diesel) and < 5 kWh of electricity consumption per tonne of bauxite extracted [26].

##### Refining/smelting

The global average energy use for the electrolysis cell is 13.4 kWh per kg of aluminium produced. If rectifiers and other cell auxiliaries, such as pollution control equipment, are included, the global average increases to 14.2 kWh per kg of aluminium produced [35], based on IAI data.

##### Process heat

The Bayer process is the most energy-intensive process in primary aluminium production. The energy consumption by the Bayer process varies at 7–21 GJ/tonne [34]. However, the aluminium industry is moving towards more energy-efficient primary production methods. One 2018 study of Columbian aluminium-producing companies showed that energy intensity can be reduced by changing the core elements of the process, including the size, processes, and temperature of the furnaces [37]. The study suggested that energy consumption could be reduced by 32% by installing an oxy-combustion technology, which pre-heats the combustion air. Costs related to thermal energy could be reduced by 50.5% per tonne of aluminium. However, the investment cost (purchase) of the technology is high, which hinders its widespread application [37].

#### Global aluminium production and energy intensity projections

The projections for the overall increase in global aluminium production are driven by technology shifts, including in lightweight vehicles and the mounting and framing equipment used for solar photovoltaic (PV) panels and large reflectors for concentrated solar power plants [38]. The assumed ratio of primary to secondary aluminium is vital in the calculation of the energy demand, because secondary aluminium production is significantly less energy intensive than primary production.

We have estimated the global aluminium energy demand to 2050 based on the projected aluminium production and energy intensity. The IEA Sustainable Development Scenario projects an annual growth rate of around 1.2% until 2030, and 15% overall growth in production between 2018 and 2030 [38]. This is a projected overall increase in global aluminium production from the current 85 million tonnes per year to just under 150 million tonnes per year. Table 6 shows the projected global aluminium production. The global recycling rate is projected to increase from 32% in 2019 currently to 45% or higher in 2050 [39]. The increased recycling rate will lead to a significant decoupling of global bauxite and alumina production from global aluminium production. Bauxite is refined to alumina (an aluminium oxide compound), and this intermediate is melted down in a further processing step to generate the end-product, aluminium. The efficiency ratio of bauxite to alumina is projected to increase from 40 to 45% which will lead to a reduction of energy demand.

**Table 6:** Assumed global aluminium market development

Parameter	[Unit]	2019	2025	2030	2035	2040	2050
Global aluminium production	[Mt/yr]	84	96	106	117	127	147
Global primary aluminium production	[Mt/yr]	63.7	68	71	73	76	81
Global aluminium recycling (data includes old scarp only, excluding new scarp –re-melted material as part of primary aluminium production)	[Mt/yr]	20.4	28	36	43	51	66
Calculated annual growth rate of the global aluminium market			2.10%	1.90%	1.74%	1.60%	1.38%
Global bauxite mining (estimate is based on aluminium growth projection)	[Mt/a]	325	368	403	438	473	543
Global alumina refineries/production	[Mt/a]	130	137	139	141	143	147
Bauxite-to-alumina ratio	[%]	40%	41%	41%	42%	43%	45%
Alumina-to-primary-aluminium ratio	[%]	49%	50%	51%	52%	53%	55%
Global: primary aluminium production share of total production	[%]	68%	66%	64%	62%	59%	55%
Global: secondary aluminium production Share of total production	[%]	32%	34%	36%	39%	41%	45%

*Secondary aluminium production* occurs through recycling schemes, after which the aluminium is re-melted and refined. The energy consumption involved is much lower than for the primary production of aluminium [35]. The aluminium sector distinguishes between new or pre-consumer scrap and old or post-consumer scrap (discarded aluminium products). Of the 33 million tonnes of aluminium recycled in 2019, 20 million tonnes was from old scrap and 14 million tonnes was from new scrap, and the share of new scrap is expected to reach 24 million tonnes in 2050 [40].

The projected energy intensities for bauxite mining and aluminium production are shown in Table 7. The fuel demand per tonne of mined bauxite mainly comprises the fuel consumed by mining vehicles. The electricity and process heat demand projections for primary and secondary aluminium reflect the improvements in the industry’s efficiency in the past decade and assume incremental improvements based on the efficiency assumptions and opportunities noted above, but with no disruptive new production technologies.

**Table 7:** Assumed energy intensities for bauxite mining and aluminium production processes

Parameter	[Unit]	2019 [estimated]	2025	2030	2035	2040	2050
Energy intensities							
Bauxite mining—energy intensity	[PJ/Mt]	0.721	0.705	0.689	0.674	0.659	0.630
Bauxite mining—fuels for mining machinery (currently 1.5 kg of fuel oil per tonne) bauxite/fuel oil = 45.6 kg/MJ	[PJ/Mt]	0.068	0.068	0.068	0.068	0.068	0.068
Bauxite mining—alumina electricity demand	[PJ/Mt]	0.653	0.637	0.621	0.605	0.590	0.561
see above in TWh	[TWh/Mt]	0.181	0.177	0.172	0.168	0.164	0.156
Bauxite mining and alumina refining—thermal energy	[PJ/Mt]	10.02	9.77	9.53	9.29	9.06	8.61
Primary aluminium—electricity (anode, electrolysis + ingot)	[PJ/Mt]	55.8	54.4	53.1	51.7	50.4	47.9
see above in TWh	[TWh/Mt]	15.5	15.1	14.7	14.4	14.0	13.3
Primary aluminium—thermal (anode, electrolysis + ingot)	[PJ/Mt]	18.4	17.9	17.5	17.1	16.6	15.8
Secondary aluminium—electricity (anode, electrolysis + ingot)	[PJ/Mt]	2.8	2.7	2.7	2.6	2.5	2.4
See above in TWh	[TWh/Mt]	0.8	0.8	0.7	0.7	0.7	0.7
Secondary aluminium—thermal (anode, electrolysis + ingot)	[PJ/Mt]	0.9	0.9	0.9	0.9	0.8	0.8

The IEA in 2020, has reported improvements in the energy efficiency (− 3% annually) of alumina refining and aluminium smelting between 2010 and 2018 [38]. These have been due to the highly energy-efficient production in China. Further reductions in global energy intensity (1.2% annually) are required under the IEA Sustainable Development Scenario, which can be achieved through a shift towards increasing rates of aluminium recycling (Table 7). Secondary production must reach 40% by 2030, with a minimum proportion from old scrap of 70% [38]. The IAI projection to 2050, with maximum recycling rates, is 43% secondary production, but material recycled from old scrap does not exceed 70% [39].

The production and energy intensity data for the aluminium sector were used to calculate the sectoral decarbonization pathway presented in the final sections of this paper.

### Steel Industry (1510 4010)

Steel is an important material for engineering and the construction sector worldwide, and is also used for everyday appliances at the domestic and industrial levels. About 52% of steel usage is for buildings and infrastructure: 16% is used for mechanical equipment, such as construction cranes and heavy machinery; 12% is used for automotives (road transport); 10% is used for metal products, including tools; 5% is used for other means of transport, including cargo ships, aeroplanes, and two-wheeler vehicles; 3% is used for electrical equipment; and 2% is used for domestic appliances, such as white goods [41].

This section provides an overview of global steel production. Table 8 shows the data for global crude steel production. The World Steel Association production data published in *2020 World Steel in Figures* is not complete for all countries, but is complete for North America (119.2 Mt) and EU 28 (150.2 Mt) (note: Bulgaria, Croatia, and Slovenia are not included in the report; [41] [42, 43].

**Table 8:** Global crude steel production data by country (million tonnes/yr)

	2017	2018	2019 [43] Prediction + 3.4%)	2020
European Union (28)	168.5	167.7	173.4	179.2
Other Europe	42.2	42.4	43.9	
Commonwealth of Independent States C.I.S	101.6	102.1	105.6	
North America (Canada, USA, Mexico)	114.8	120.3	124.3	100.5
Caribbean	0.6	0.6	0.6	
South America	44.1	44.9	46.5	
Africa	14.8	17.4	18.0	
Middle East	34.5	38.0	39.3	
Asia	1205.5	1278.0	1321.5	
Oceania	6.0	6.3	6.6	
World (Mt)	1732.2	1816.6	1878.4	1878

#### Primary and secondary steel production

There are different routes by which steel is produced. Crude or primary steel is produced from iron ore and secondary steel is produced from recycled steel. These two routes use different technologies and different energy sources. The share of secondary steel production increased by 25% globally in 2013, and by 28% in 2018 [43]. Secondary steel production is limited by scrap availability. Currently, the total global scrap steel collection rate is 85% [41], i.e., on average, 85% of steel consumed or utilized will be collected and recycled [44]. The scrap collection rate varies for different steel applications: for structural reinforcement, it is as low as 50%, whereas for industrial equipment, it is as high as 97% [41]. Altogether, scrap inputs account for about 35% of total primary steel production. Secondary steel production is up to 74% less energy intensive than making steel from iron ore (primary production) [45]. By 2030, this share should increase to 40% under the IEA Sustainable Development Scenario [41]. The share of scrap in primary steel production varies among countries and from year to year (Table 9).

- In the **EU 28**, the proportion of recycled steel in crude steel production was 55.9% in 2018.
- In the **USA**, the proportion of steel scrap in crude steel production was 69.4% in 2018.

**Table 9:** Share of scrap (%) in crude steel production, by region, 2018

	2018 (%)	Change from previous year (%)
EU 28	55.9	+ 0.3
USA	69.4	+ 2.2
Japan	35	+ 2.1
Russia	42.5	+ 5.5
Turkey	80.7	− 0.4
South Korea	41.4	− 2.3

*Source* [46]

**Fig. 1:**
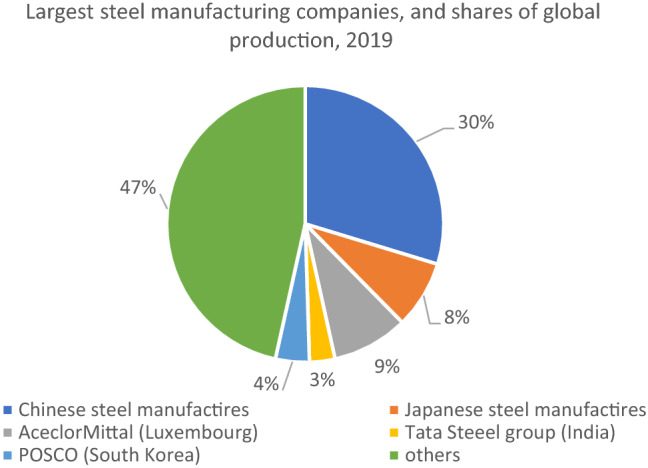
Largest steel manufacturing companies, and shares of global production, 2019

Global steel production is highly concentrated, and 12 companies are responsible for > 50% of global steel production. Steel companies with headquarters in China dominate the sector (Fig. 1). Seven corporations based in China are responsible for 30% of global steel production. European steel manufacturers produce 9% of global steel, Japanese companies 7%, South Korean companies 4%, and Indian steel manufacturers 3%.

The dominant role of Chinese manufacturers in the global market explains the increase in global production because demand is high and there have been improvements in energy efficiency at the global scale.

*Impact of COVID-19 on global steel production* Global crude steel production decreased by 1.4% in the first 3 months of 2020 compared with that in the same period of the previous year, and in March, a decrease of 6% was reported [47]. The largest decline in steel production in the first quarter of the year (Q1) occurred in the EU (− 10%), Japan (− 9.7%), South Korea (-7.9%) and North America (− 4%) [47]. The long-term consequences of COVID-19 on the steel sector are unclear. During the Global Financial Crisis (GFC) in 2009, steel production in Europe alone dropped by 30% compared with that in previous years.

#### Technological overview of steel production

On average, 20 GJ of energy is consumed to produce one tonne of crude steel globally [48]. The IEA’s Tracking Industry Report (IEA, 2020d) showed a gradual decline in energy intensity between 2009 and 2018. The largest year-to-year fall was in 2017–2018, when energy intensity declined by 3.6%. As mentioned earlier, there are two routes by which steel is produced (Table 10). Primary or crude steel is produced by the coal- or natural-gas-based Blast furnace–basic oxygen furnace (BF–BOF) route, in which iron ore is reduced at very high temperatures in a blast furnace. Iron ore is melted to a liquefied form (pig iron or direct reduced iron [DRI]), and then oxidized and rolled (Table 11). Coal or natural gas are required to generate high temperatures of up to 1650 °C. In the secondary production route, scrap steel is melted in Electric arc furnaces (EAFs). The EAF route has the lowest emissions intensities. In the EAF (gas-fuelled) process, scrap is usually blended at a rate of about 10% with DRI. A more energy-efficient pathway for primary production is to use scrap steel with ore-based inputs in BF–BOF production, usually at a rate of 15%–20% [43].

**Table 10:** Steel production—main processes

	Blast furnace–basic oxygen furnace (BF–BOF); 75% of steel is made with this process		Electric arc furnace (EAF); 25% of steel is made with this process	
Energy indirectly consumed (mining, preparation, and transport of raw materials)	9%		6%	
Energy input from	Application as energy in:	Energy use (%) in total process	Application as energy in:	Energy use (%) in total process
Coal	Blast furnaces (BFs), sinter, and coking plant	89%	Coke production, BF pulverised coal injection	11%
Electricity	EAFs, rolling mills, and motors	7%	Melting of steel scrap	50%
Natural gas	Furnaces, power generators	3%	BF injection, DRI production	38%
Other gases and sources (%)	Steam production	1%	BF injection	1%

*BF–BOF* production of primary steel from iron ore (oxygen is blown through liquid pig iron, increasing its temperature and releasing carbon), *EAF* production of secondary steel from scrap metal

**Table 11:** Steel production—main processes and energy requirements

Process	Energy use (GJ/metric tonne)			
	Absolute minimum	Practical minimum	Actual average requirement	% over practical minimum
Liquid metal ‘pig iron’ (iron ore is reduced to iron)	9.8	10.4	13.5	23
Liquid hot metal: basic oxygen furnace (iron ore is converted to steel)	7.9	8.2	11	25
Liquid hot metal: electric arc furnace	1.3	1.6	2.25	29
Hot rolling flat (after rolling, steel is delivered as strips, plates, bars, etc.)	0.03	0.9	2.2	59
Cold rolling flat	0.02	0.02	1.2	98

##### Emissions benchmarks for the steel industry

Table 12 shows the emissions values allowed for the manufacture of steel under the emission trading scheme of the European Union (EU-ETS). The manufacture of secondary steel by electric arc furnaces (EAFs) is significantly less carbon intensive – in tonne CO_2_ per tonne of steel (tCO_2_/tonne)—than the production of primary steel by the iron-ore-based route, in which hot metal is produced in blast furnaces (blast furnace–basic oxygen furnace [BF–BOF] route).

**Table 12:** EU-ETS benchmark values for iron and steel manufacture as of February 2020

Material	Benchmark (tCO_2_e/tonne product)
Hot metal	1.328
Sintered ore	0.171
Iron casting	0.325
Electric arc furnace (EAF) high-alloy steel	0.352
Electric arc furnace (EAF) carbon steel	0.283
Coke (excluding lignite coke)	0.286

*Source* [49]

#### Projections for the global steel industry

To calculate the future energy demand for the global steel industry requires a range of assumption—from the actual market volume, to recycling rates and energy intensities, to the actual production process itself. Unlike the aluminium industry, steel manufacturing involves GHG emissions that are not related to energy generation but to the process itself. The emissions intensity of the steel sector, and specifically steel plants, depends upon the production route (BF–BOF or EAF) and the energy source (Table 13). Both routes can, for example, be fuelled by natural gas [43]. The actual process emissions per tonne for each of the production process options are assumed to remain at current levels. Table 13 shows the most important assumptions made for the development of the global steel industry:

1. The development of the global sales markets in tonnes per year.
2. The share of recycled steel the global steel market.
3. The assumed technical development of primary and secondary steel production in terms of the specific total energy requirements (in GJ/tonne steel), both in regard to electricity and process heat demand.
4. The development of the market shares of the three main production processes for steel. Each production process has process-typical emissions that are independent of the energy supply.

**Table 13:** Assumed market and energy intensity developments for the global steel industry according to the production process

Parameter	[Unit]	2019	2025	2030	2035	2040	2050
Assumed global steel production volumes							
Global iron ore production—estimates based on steel growth projections	[Mt/yr]	2339	2377	2511	2676	2851	3289
Global: annual production volume—iron and steel industry	[Mt/yr]	1869.6	1904.2	2018.4	2159.7	2310.9	2695.4
Calculated annual growth rate for global steel market	[%/yr]		0.95%	1.13%	1.31%	1.31%	1.48%
Development of production structure (primary and secondary)							
PRIMARY steel production	[%]	65%	63%	61%	59%	56%	52%
SECONDARY steel production (share of scrap)	[%]	35%	37.2%	39.3%	41.5%	43.7%	48%
Share of electricity in PRIMARY steel production	[%]	2%	2%	2%	2%	2%	2%
Share of electricity in SECONDARY steel production	[%]	91%	91%	91%	91%	91%	91%
Energy intensities							
Energy intensity for Iron Ore Mining	[PJ/Mt]	0.069	0.067	0.066	0.064	0.062	0.059
Global: average energy intensity for steel production	[GJ/tonne]	18.6	12.81	12.4	12.2	12.0	11.4
Global range: average energy intensity for PRIMARY steel production	[GJ/tonne]	21	16	16	16	16	16
Global range: average energy intensity for SECONDARY steel production	[GJ/tonne]	9.1	8.26	7.65	7.55	7.45	7
Primary steel production—electricity demand	[GJ/tonne]	0.42	0.31	0.31	0.31	0.31	0.31
Primary steel production—process heat demand	[GJ/tonne]	15.57	11.51	11.51	11.51	11.51	11.51
Secondary steel production—electricity demand	[GJ/tonne]	8.28	7.52	6.96	6.87	6.78	6.37
Secondary steel production—process heat demand	[GJ/tonne]	6.75	6.13	5.68	5.61	5.53	5.20
Electricity intensities							
Electricity intensity—PRIMARY steel production	[TWh/Mt]	0.12	0.09	0.09	0.09	0.09	0.09
Electricity intensity—SECONDARY steel production	[TWh/Mt]	2.30	2.09	1.93	1.91	1.88	1.77
Development of process-related emissions							
Specific process emissions—assumption in the OECM for the global average)	[t CO_2_/tonne crude steel]	1.06	0.92	0.60	0.37	0.23	0.08
Basic oxygen furnace (BOF)—production share	[%]	65%	58%	35%	20%	10%	0%
Basic oxygen furnace (BOF)—emission factor	[t CO_2_/tonne steel]	1.46	1.46	1.46	1.46	1.46	1.46
Open hearth furnace (OHF)—production share	[%]	5%	3.0%	2.5%	1.0%	1.0%	0%
Open hearth furnace (OHF)—emission factor	[t CO_2_/tonne steel]	1.72	1.72	1.72	1.72	1.72	1.72
Electric arc furnace (EAF)—production share	[%]	30%	40%	63%	79%	89%	100%
Electric arc furnace (EAF)—emission factor	[t CO_2_/tonne steel]	0.08	0.08	0.08	0.08	0.08	0.08

The entire calculated energy requirement of the steel industry, as well as the energy-related and process-related CO_2_ emissions, is the result of these—directly dependent—assumptions.

The global steel market is estimated to grow by 1%–1.5% throughout the entire modelling period. The recycling rates are assumed to increase so that the share of secondary steel will grow from 35% in 2019 to 48% in 2050. The shares of electricity for primary and secondary steel in the overall production process are projected to remain at the current levels. Secondary steel production is, to a large extent, based on electricity, whereas primary steel production is 98% dependent upon process heat for melting processes. The energy and electricity intensities per tonne of manufactured volume for both secondary and primary steel production are based on IEA projections [43]. Table 13 shows all the assumed market and energy intensity developments for the global steel industry according to the production process.

## Results: global energy demand projections for GICS-1510-classified industries

Based on the assumed global market development for the aluminium, steel, and chemical industries and the increased energy efficiencies for all parts of the production processes (as documented), the demands for electricity, heating, and fuels have been calculated with the OECM. The projected energy efficiencies for all three industries are ambitious, and the recycling estimates for aluminium and steel are relatively high. However, in this research, we did not look into alternative products for aluminium or steel or whether all the products produced by the chemical industry are sustainable. The implementation of a circular economy, reduced packaging, alternative materials to plastics, and an overall reduction in the consumption of short-lived products will lead to a reduced market volume for the chemical industry, and less materials will be used. The use of plastics is directly associated with the non-energy use of oil, and a reduction in the plastic demand will significantly reduce the energy demand of the petrochemical industry. Therefore, the projected energy demands only represent possible trajectories under assumptions of technical efficiency, but exclude any lifestyle changes.

### Demand: electricity and process heat by industry

Table 14 shows the electricity and process heat demands for the aluminium, steel, and chemical industries, calculated on the basis of the documented assumptions, from the base year 2019 to the end of the scenario time-frame in 2050. In 2019, the largest electricity consumer of the three sub-sectors analysed was the chemical industry, with around 1500 TWh/yr, closely followed by the steel industry, with 1250 TWh/yr, and the aluminium industry, with 1000 TWh/yr. Combined, this represented 40% of the global industry electricity demand or 16% of the total global electricity demand (Table 15). The overall electricity demand will increase significantly until 2050: both the chemical and steel industries are projected to double their electricity consumption, while the aluminium industry will increase its consumption by around 10%. The increased electricity demands in the steel and chemical industries will arise from process heat electrification, instituted to reduce fossil fuel use (and associated GHG emissions) for heat generation.

**Table 14:** Projected electricity and process heat demands for the aluminium, steel, and chemical industries to 2050

Sub-sector	Unit	2019	2025	2030	2035	2040	2050
Aluminium industry							
Total electricity demand—aluminium industry	[PJ/yr]	3694	3860	3924	3982	4035	4125
Total electricity demand—aluminium industry (including re-melting)	[TWh/yr]	1026	1048	1066	1082	1097	1123
Electricity demand—primary aluminium	[TWh/yr]	1005	1027	1040	1051	1062	1079
Electricity demand—secondary aluminium (excluding re-melting)	[TWh/yr]	21	21	26	31	36	44
Total process heat demand—aluminium industry	[PJ/yr]	3110	2581	2590	2597	2601	2601
Process heat demand—primary aluminium	[PJ/yr]	3079	2556	2559	2560	2558	2549
Process heat demand—secondary aluminium	[PJ/yr]	31	25	31	37	42	52
Heat demand < 100 °C	[PJ/yr]	261	216	217	218	218	218
Heat demand 100–500 °C	[PJ/yr]	48	40	40	40	40	40
Heat demand 500–1000 °C	[PJ/yr]	569	472	474	475	476	476
Heat demand > 1000 °C	[PJ/yr]	2232	1852	1859	1864	1867	1867
Steel industry							
Total electricity demand—iron & steel industry	[PJ/yr]	4559	5691	5906	6550	7245	8676
Total electricity demand—iron & steel industry	[TWh/yr]	1266	1581	1641	1819	2012	2410
Electricity demand—primary steel	[TWh/yr]	83	103	105	109	112	121
Electricity demand—secondary steel	[TWh/yr]	1184	1478	1535	1711	1900	2289
Total process heat demand—iron & steel industry (final energy)	[PJ/yr]	17,451	18,146	18,639	19,603	20,604	22,900
Process heat demand—primary steel	[PJ/yr]	13,269	13,797	14,120	14,569	15,011	16,163
Process heat demand—secondary steel	[PJ/yr]	4183	4349	4518	5034	5593	6738
Heat demand	[PJ/yr]	13,060	18,146	18,639	19,603	20,604	22,900
Heat share:	[%]	74%	76%	76%	75%	74%	73%
Heat demand < 100 °C	[PJ/yr]	595	2341	2405	2529	2658	2955
Heat demand 100–500 °C	[PJ/yr]	211	336	345	363	382	424
Heat demand 500–1000 °C	[PJ/yr]	2489	5038	5175	5442	5720	6358
Heat demand > 1000 °C	[PJ/yr]	9765	10,431	10,714	11,268	11,844	13,164
Chemical industries							
Chemical industry—electricity demand by sub-sector							
Pharmaceutical industry	[PJ/yr]	1431	1652	1873	2118	2341	2799
	[TWh/yr]	398	459	520	588	650	778
Agricultural chemicals	[PJ/yr]	782	899	1,019	1,152	1,274	1,523
	[TWh/yr]	217	250	283	320	354	423
Inorganic chemicals and consumer products	[PJ/yr]	1447	1663	1884	2131	2355	2817
	[TWh/yr]	402	462	523	592	654	782
Manufactured fibres & synthetic rubber	[PJ/yr]	273	314	356	403	445	532
	[TWh/yr]	76	87	99	112	124	148
Bulk petrochemicals & intermediates, plastic resins	[PJ/yr]	1450	1649	1849	2070	2264	2651
	[TWh/yr]	403	458	514	575	629	736
Total chemical industry	[PJ/yr]	5384	6178	6981	7874	8678	10,323
	[TWh/yr]	1496	1716	1939	2187	2411	2867
Heat demand	[PJ/yr]	12,163	15,949	18,024	20,329	22,406	26,653
Heat share:	[%]	56%	56%	56%	56%	56%	56%
Heat demand < 100 °C	[PJ/yr]	2196	2879	3254	3670	4044	4811
Heat demand 100–500 °C	[PJ/yr]	2722	3570	4034	4550	5015	5965
Heat demand 500–1000 °C	[PJ/yr]	5813	7623	8615	9716	10,709	12,739
Heat demand > 1000 °C	[PJ/yr]	1432	1878	2122	2394	2638	3138

**Table 15:** Total global electricity demand for the aluminium, steel and chemical industries

	Unit	2019	2025	2030	2035	2040	2050
Total electricity demand	[PJ/yr]	13,637	15,728	16,811	18,405	19,957	23,123
Aluminium, steel, and chemical industries	[TWh/yr]	3788	4345	4646	5089	5520	6400
Share of global electricity demand	[%]	16%	20%	22%	22%	23%	21%

To calculate the 1.5 °C pathways based on the renewable energy supply, it is necessary to separate the temperature levels for process heat because not all renewable energy technologies can produce high temperature levels. Whereas the heat generation for low heat levels can be achieved with renewable-electricity-supplied heat pumps, temperatures over 500 °C are assumed to be generated by predominantly combustion processes based on bio-energy up until 2030. After 2030, the share of electric process heat from resistance and electric arc furnaces is projected to increase to replace fossil fuels. Hydrogen and synthetic fuels will also play increasing roles in supplying high-temperature process heat. Table 16 shows the total process heat demands by temperature level for all three industries analysed combined. The overall heat demand of these sectors represented 20% of the global heat demand in 2019. This share is projected to increase to 37% due to a significant reduction in the heat demand in the building sector [50]. The steel and chemical industries had similar process heat demands in 2019, of 13 EJ/yr and 12 EJ/yr, respectively. In comparison, the process heat demand of the aluminium industry was a quarter of this, at 3 EJ/yr.

**Table 16:** Total global process demand, by temperature level, in the aluminium, steel, and chemical industries

	Unit	2019	2025	2030	2035	2040	2050
Total (process) heat demand Aluminium, steel, and chemical industries	[PJ/a]	32,725	36,676	39,253	42,530	45,611	52,154
Share of global (process) heat demand	[%]	20%	22%	24%	27%	30%	37%
Share of global (process) heat demand by temperature level							
Heat demand < 100 °C		14%	28%	27%	26%	25%	23%
Heat demand 100–500 °C		26%	48%	47%	46%	45%	44%
Heat demand 500–1000 °C		62%	66%	65%	65%	64%	64%
Heat demand > 1000 °C		53%	44%	42%	40%	39%	37%

The calculation of process heat has been broken down into four temperature levels: low (< 100 °C), medium low (100–500 °C), medium high (500–100 °C), and high (> 1000 °C). Most of the process heat required by the aluminium industry is high-temperature heat (72%), whereas the steel industry requires only 57% high-temperature heat. The majority of the process heat required by the chemical industry is in the medium–high level (48%), between 500 °C and 1000 °C (Table 16).

#### Demand: final energy

The aluminium, steel, and chemical industries combined accounted for 34% of the global industrial final energy demand and 11% of the total final energy demand in 2019 (Table 17). The combined energy share of these three sectors will increase to 41% by 2050 under the OECM scenario in response to the assumed higher efficiencies in other industry sectors, such as construction and mining. The overall energy demand of the three sectors will increase from 41 EJ/yr to 72 EJ/yr, driven mainly by the projected increase in the global GDP and therefore the production volume.

**Table 17:** Total global final energy demand in the aluminium, steel, and chemical industries

	Unit	2019	2025	2030	2035	2040	2050
Total final energy demand Aluminium, steel, and chemical industries	[PJ/yr]	41,434	48,312	51,922	56,820	61,660	72,172
Share of global final energy INDUSTRY demand	[%]	34%	41%	40%	41%	42%	41%
Share of global final energy demand	[%]	11%	14%	15%	17%	19%	22%

### Supply: electricity and process heat under a 1.5 °C pathway

The supply side of this 1.5 °C energy scenario pathway builds upon modelling undertaken in an interdisciplinary project led by the University of Technology Sydney. The project modelled sectoral and regional decarbonization pathways to achieve the Paris climate goals—to maintain global warming well below 2 °C and to “pursue efforts” to limit it to 1.5 °C. That project produced the One Earth Climate Model (OECM), a detailed bottom-up examination of the potential to decarbonize the energy sector. The results of this ongoing research were published in 2019 [7], 2020 [51], and 2021 [50]. For this analysis, the 1.5 °C supply scenario has been updated and more industry sectors have been included. Table 18 shows the development of the projected global electricity generation. Coal- and lignite-based power plants will be phased out first, followed by gas power plants (the last fossil-fuelled power generation technology) after 2040. Renewable power plants, especially solar PV, and onshore and offshore wind, are projected to have the largest growth rates, leading to a combined share of 70% of global electricity generation by 2050. The overall renewable electricity share will increase from 25% in 2019 to 74% in 2030 and to 100% by 2050, with the full decarbonization of the power sector. Specific CO_2_ emissions per kilowatt-hour will decline from 509 to 136 g by 2030.

**Table 18:** Electricity supply under the OECM 1.5 °C pathway

		2019	2025	2030	2035	2040	2050
Coal	[%]	31	17	5	1	0	0
Lignite	[%]	7	1	1	1	0	0
Gas	[%]	24	20	15	8	4	0
Oil	[%]	3	2	1	0	0	0
Nuclear	[%]	10	7	4	2	0	0
Hydrogen (produced with renewable electricity)	[%]	0	0	0	2	2	5
Hydro power	[%]	16	14	13	10	9	9
Wind	[%]	5	14	22	28	32	36
PV	[%]	2	18	30	37	36	34
Biomass	[%]	1	3	2	2	1	1
Geothermal	[%]	0	1	2	2	3	3
Solar thermal power plants	[%]	0	1	4	8	10	10
Ocean energy	[%]	0	0	0	1	1	1
Renewable share	[%]	25	52	74	89	95	100
Specific CO_2_ emissions per kWh (according to OECM 1.5˚C scenario)	[gCO_2_/kWh]	509	290	136	53	24	0

The process heat supply in 2019 relied heavily on fossil fuels (83%), mainly coal (33%) and gas (36%). Renewables played a minor role and the majority of renewable process heat was from biomass. Table 19 shows the assumed trajectory for the generation of industry process heat. To increase the renewable energy shares—especially for the high temperature level—is more challenging than for the electricity sector. A fuel switch from coal and gas to biomass requires less technical changes than a transition towards geothermal energy, all forms of heat pumps, or direct electricity use [52]. The OECM assumes that the global limit for sustainable biomass is around 100 EJ per year [53]. The generation of high-temperature heat requires concentrated solar thermal plants. However, solar thermal process heat is limited to low temperatures in most regions because concentrated solar plants require direct sunlight with no cloud coverage and can therefore only operate in the global sunbelt range in most regions [54]. Therefore, it is assumed that process heat will be increasingly derived from electricity-based technologies: heat pumps for the low temperature levels, and direct resistance electricity and electric arc furnaces for the medium and high temperature levels. However, to implement appliances to generate electricity-based process heat will require significant changes in the production process. A significant increase in this technology is assumed to be unavailable before 2025, but will increase rapidly between 2026 and 2030. Hydrogen and synthetic fuels produced with renewable electricity will increase after 2030, especially for processes that cannot be electrified. For details, see Table 17.

**Table 19:** Heat supply under the OECM 1.5 °C pathway

Industry process heat supply—incl. industry combined heat and power (CHP)	Unit	2019	2025	2030	2035	2040	2050
Coal	[%]	33	18	11	6	0	0
Oil	[%]	14	5	3	1	0	0
Gas	[%]	36	38	25	22	17	0
Renewable heat (bio-energy, geothermal, and solar thermal)	[%]	9	24	32	27	21	25
Electricity for heat	[%]	1	8	22	36	49	60
Heat (district)	[%]	7	6	6	7	7	7
Hydrogen and synthetic fuels	[%]	0	0	1	2	6	8

### Global CO_2_ emissions and carbon budget: aluminium, steel, and chemical industries

In the last step, we calculated the energy-related carbon emissions. The OECM 1.5 °C Net-Zero pathway is based on efficient energy use and a renewable energy supply only, leading to full energy decarbonization by 2050. No negative emission technologies are used and the OECM results in zero energy-related carbon emissions. However, process emissions are compensated by nature-based solutions, such as increased forest coverage. The details of the analysis of non-energy-related GHG emissions, which includes the emissions from land-use changes (AFOLU), chemical substances, and aerosols, are documented by [55].

The total carbon budget to achieve a temperature increase of 1.5 °C with 67% likelihood is 400 GtCO_2_ between 2020 and 2050, as defined by the Intergovernmental Panel on Climate Change [1]. The OECM calculated all the energy uses across all industry sectors, buildings, and transport. However, we have focused on three main industries in this paper. The basic requirement for compliance with the CO_2_ pathways shown in Table 20 is the successful global introduction of electricity from renewable energies and the simultaneous phase-out of coal in OECD countries by 2030 and in all other countries, including China and India, by 2040. Whereas the energy-efficiency measures and the implementation of new process heat technologies are tasks of the aluminium, steel and chemical industries, the decarbonization of the electricity supply is to a large extent the responsibility of the utilities sector.

**Table 20:** Energy-related CO_2_ emissions—aluminium, steel, and chemical industries

	Unit	2019	2025	2030	2035	2040	2050
Aluminium industry							
CO_2_ emissions—power supply	[Mt CO_2_/yr]	522	305	145	57	26	0
CO_2_ emissions—heat supply	[Mt CO_2_/yr]	191	108	68	47	24	0
CO_2_ emissions—total energy supply	[Mt CO_2_/yr]	713	413	213	104	50	0
Process-related emissions	[Mt CO_2_/yr]	210	229	240	250	258	270
Specific energy-related CO_2_ emissions per tonne aluminium	[tCO_2_/tonne aluminium]	4.8	3.5	2.9	2.5	2.2	1.8
Specific process-related CO_2_ emissions per tonne aluminium	[tCO_2_/tonne aluminium]	2.5	2.4	2.3	2.1	2.0	1.8
Steel industry							
CO_2_ emissions—power supply	[Mt CO_2_/yr]	645	459	223	96	48	0
CO_2_ emissions—heat supply	[Mt CO_2_/yr]	1073	762	489	353	187	0
CO_2_ emissions—total energy supply	[Mt CO_2_/yr]	1717	1221	712	449	235	0
Process-related emissions	[Mt CO_2_/yr]	1980	1757	1219	804	542	216
Specific energy-related CO_2_ emissions per tonne steel	[tCO_2_/tonne steel]	0.6	0.4	0.2	0.2	0.1	0.0
Chemical industry							
CO_2_ emissions—power supply	[Mt CO_2_/yr]	761	499	264	115	58	0
CO_2_ emissions—heat supply	[Mt CO_2_/yr]	1,257	994	707	554	323	0
CO_2_ emissions—total energy supply	[Mt CO_2_/yr]	2,019	1,492	971	669	380	0
Chemical Industry total non-energy GHG	[MtCO_2_ eq./yr]	2,520	1,852	1,220	991	775	682
Total: aluminium, steel, and chemical industries							
Total CO_2_ emitted by the aluminium, steel, and chemical industries	[Mt CO_2_/a]	3,741	2,717	1,686	1,120	617	2
Share of global energy-related CO_2_ emissions	[%]	11	11	10	12	13	0

**Fig. 2:**
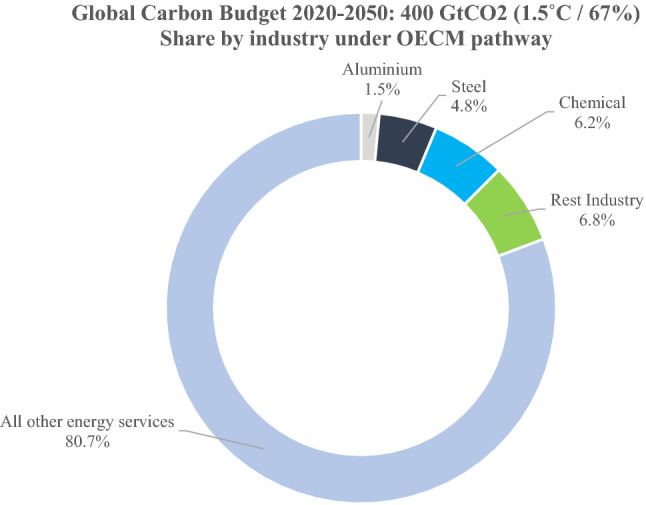
Global cumulative energy-related CO_2_ budget (2020–2050)—shares for the aluminium, steel, and chemical industries

The shares of the cumulative carbon budget required to achieve the 1.5 °C Net-Zero target are shown in Fig. 2. The total energy-related CO_2_ for the aluminium industry between 2020 and 2050 is calculated to be 6.1 Gt, 1.5% of the total budget. The steel industry will emit 19.1 Gt CO_2_ or 4.8%, and the chemical industry will emit the highest share of 24.8 GtCO_2_ or 6.2% of the total carbon budget. The remaining industries will emit 27.1 GtCO_2_ (6.8%), whereas all other energy-related activities will emit 323 GtCO_2_, or 80.7% of the budget.

## Conclusions, limitations, and further research

The development of energy and emissions pathways for industry sectors requires an energy model with high technical resolution. Compared with regional and global energy scenarios, sectorial pathways for industries are based on significantly more statistical data and must be developed in close co-operation with industry partners.

Furthermore, the estimation of carbon budgets for specific industry sectors based on GICS requires a holistic approach, and all sectors must be considered in order to capture the interactions between the different industries and the energy sector. To estimate a carbon budget based on current emissions for a single branch, such as the aluminium industry, will inevitably lead to inaccurate results because this approach does not consider the possible technical developments in other individual industries. The current discussions of net-zero targets for specific industries are often developed for a single industrial sector in isolation. This means that the total of all sub-concepts for certain industries might exceed the actual CO_2_ emitted, and/or the responsibility for the reduction of CO_2_ may be shifted to other sectors.

In this research, bottom-up energy demand projections for the chemical, aluminium, and steel industries formed the basis for the supply scenarios for electricity, process heat, and fuels. The supply of carbon-free electricity is the key to the decarbonization of all three industry sectors. Furthermore, the electricity demand will increase with the electrification of process heat to replace fuels. Power utilities will therefore play a crucial role in reaching the decarbonization targets of those industries. The decarbonization of process heat requires changes in the production process, and is therefore the core responsibility of the industry itself.

We found that it is technically possible to decarbonize the energy supply of the three industries analysed on the basis of available technologies. However, the OECM 1.5 °C pathway is not a prognosis, but a back-casting scenario that shows what must be done to achieve the carbon target. More-detailed analyses for specific industry locations, e.g., China or India, are required because our global analysis simply processes and calculates energy demand projections on the basis of average global energy intensities. Moreover, energy demand was calculated with energy intensities (e.g., for steel production) derived with a literature search. Energy statistics, especially for the chemical industry, are scarce and all energy demands sub-sectors are based on GDP projections.

More research is required for industries in specific GICS classes, in terms of both statistical data and the current and future energy intensities for industry-specific processes. A central database of energy intensities and energy demands for each GICS class would significantly enhance the level of detail available for the calculation of net-zero pathways in the future.

## Supplementary Information

Below is the link to the electronic supplementary material.

Supplementary file1 (XLSX 342 kb)
